# The New Louisiana Quarantine

**Published:** 1885-07

**Authors:** 


### THE NEW LOUISIANA QUARANTINE.

### A VISIT OF INSPECTION BY STATE SANITARY OFFICERS.

Our readers will remember that the Louisiana Board of Health have put in operation the plan of quarantine devised, we believe, by Dr. Holt, the president, and recommended by him to the conference of State health authorities, and by them adopted in June, 1884. This plan, called by Dr. Holt "maritime sanitation," consists of thoroughly disinfecting ship and cargo, at the quarantine station, by an improved process, an interesting description of which we give below, extracted from the *Times Democrat.* It will be remembered, too, that the plan of quarantine contemplated substituting this process for *any detention whatever*, the period of five days being the limit to which any vessel was subject, and those five days being counted from time of departure of vessel from port, were necessarily spent in transit. It will be remembered, also, that the Texas health officer made a vigorous protest against this,—pointing out the danger of practically removing all restrictions from persons who, having been exposed, not only on, and before embarking, but during the voyage, if the ship or cargo be infected,—and even up to, and including the unloading of the cargo for disinfecting, and who, of course, could not be disinfected; and claiming, rightly, that the person so exposed, should be detained not less than five days from the time of last exposure. A case of yellow fever occurring in New Orleans, immediately after this protest, (which protest, it will be remembered, led to sharp correspondence between the health officers of Texas and Louisiana), and which case demonstrated clearly the correctness of Dr. Swearingen's position, caused the rules adopted by the Louisiana Board, on the subject of quarantine, to be modified, we understand, so as to cover the period of incubation by detention, as insisted on by the Texas sanitary officer.

But the improved process of disinfection, and a visit of inspection to the quarantine station below New Orleans, by the New Orleans Board and the sanitary officers of adjoining States is the subject of this article. Drs. Swearingen, of Texas, Thornton, of Memphis, and Fite, of Nashville, and other gentlemen, by invitation of the Louisiana Board of Health, visited New Orleans lately, and made an excursion down the river, on a steam tug, to witness the practical working of the new "maritime sanitation." From the account given of the trip by the *Times Democrat*, it was a trip in which "business and pleasure" were largely combined, and the gentlemen, from all accounts, had a royal time. They had feasting on such things as tenderloin trout and softshell crabs, champaigne and real Havana cigars; and toasting, and a good time generally, barring the "horn-billed bird of the bush," as the facetious quilldriver of the T. D. calls the festive mosquito, which, from his account, made "midnight hideous" and caused even Dr. Swearingen to say "unkind words." At the dinner — dinners are indispensable to a satisfactory "inspection" of a quarantine — toasts were, as usual, the order. Of this the T. D. says.

* * * "closed amidst loud applause." [Stereotyped: newspaper men keep that expression standing.—Ed.] "Dr. Swearingen, of Texas, was next called on. With reference to the new system, he said it was a practical application of all known and supposed agents for the destruction of yellow fever poison, and pointed to a higher order of things. If it failed, it would be the most splendid failure in the world's history, and would even command the admiration of all time."

"Dr. Swearingen referred to his strong belief in scientific methods, and, although guarded in his language, expressed such broad scientific views as most favorably impressed all present. His manner, his ease of diction, and his scope of intellect, produced a marked effect, as did also that of Dr. Thornton, of Tennessee."

Texas is proud of her Health Officer, and we appreciate the above handsome compliment to him.

Of the process of disinfection, the *Times Democrat* says:

"Under the old system of fumigation used here, the process was one almost primitive in its simplicity. It consisted merely of burning, in iron pots in the hold of the vessel and in the cabin, of a certain quantity of sulphur, the fumes of which were intended to do the work of destruction to the bacteria of disease. By way of disinfection, the decks, etc., were sprinkled with a solution of carbolic acid and the bilge washed out.

The new process, which is the result of long and severe study by Dr. Holt and his coadjutors of the board, has introduced entirely new features, doing away entirely with the objectionable carbolic acid, and employing instead of the untrustworthy iron sulphur pots, a more reliable mode of fumigation. As a part of the new process, the hot closet referred to was adopted.

When a vessel requiring the services of the quarantine officers arrives at the station, the first thing done is to have brought on deck every article of wearing apparel, bed clothes, table linen, and in fact every piece of textile fabric, rags and upholstery. These are exposed to the air and subjected to a thorough drenching by means of a sprinkling nozzle attached to the hose of the quarantine boat Laurel. The liquid used is a solution of the bi-chloride of mercury, one part in a thousand, of pure water. This is recognized as a most potent germicide and disinfectant, no other solution possessing the same efficacy being known that would combine the same effectiveness, and at the same time be harmless upon all fabrics.

After all the wearing apparel, etc., has been drenched, as referred to, it is then taken ashore and placed in the hot closet. This box, for it looks like a huge box, has double sides, top and bottom, and between them are several thicknesses of the heaviest felt to prevent radiation of heat. A long steam worm, which can be connected with the tug, completely covers the floor. After all the articles treated to the bi-chloride solution have been inclosed in the hot closet, steam is turned on from the tug, and the temperature inside runs up to between 212 and 270 degrees fahrenheit. This dry heat is kept up for an hour, a heat which is too great for any animal organism to survive in. The articles are then taken out and aired, being now perfectly dry and thoroughly disinfected.

This accomplished, attention is then given to the vessel itself. And here comes in another of the improvements of the present Board of Health. The large steam tug Laurel has fitted up in her stern a large iron furnace, divided into twelve compartments, each nine by twelve inches, in which fit cast iron pans three feet long. In these pans sulphur is placed, and when everything is ready for operation it is fired by placing a red hot iron in the sulphur.

As the furnace is open in the rear, it admits of a free ingress of air over the burning sulphur. From the front of the furnace there extends a large pipe connected with a revolving fan, operated by an independent engine. When the fan is set in motion it draws in a great current over the burning sulphur, which gives off sulphurous acid fumes. These the fan delivers through a discharge pipe through the roof in a volume almost inconceivable in strength. On the roof of the tug, attached to the discharge pipe, are the connections used to conduct the powerful current into the hold of the vessel.

These connections consist of galvanized iron tubing twelve inches in diameter, and from these oiled canvas hose run down to the bottom of the ship. Each compartment of a steamer is charged with the sulphurous fumes which, flowing in, soon fills every interstice between timbers, boxes or bags in the cargo, obliterating all life in a few minutes. So great is the volume of gas introduced, it requires but a few moments to fill the largest compartment. The bilges having been washed by an inflow of clear water, there is then no space left untouched by the gas, and rats, roaches and insects are stifled before they can escape."

